# Orthogonal *MET* analysis in a population‐representative stage II–III colon cancer cohort: prognostic and potential therapeutic implications

**DOI:** 10.1002/1878-0261.13089

**Published:** 2021-11-01

**Authors:** Stephanie G. Craig, Svenja Mende, Matthew P. Humphries, Victoria Bingham, Amélie Viratham Pulsawatdi, Maurice B. Loughrey, Helen G. Coleman, Stephen McQuaid, Richard H. Wilson, Sandra Van Schaeybroeck, Jacqueline A. James, Manuel Salto‐Tellez

**Affiliations:** ^1^ Precision Medicine Centre of Excellence Patrick G Johnston Centre for Cancer Research Queen's University Belfast UK; ^2^ Department of Cellular Pathology Royal Victoria Hospital Belfast Health and Social Care Trust Belfast UK; ^3^ Centre for Public Health Queen's University Belfast UK; ^4^ Institute for Cancer Sciences University of Glasgow UK

**Keywords:** c‐MET IHC protein, colon cancer, *MET* amplification, *MET* R970C mutation, *MET* RNA‐ISH, *MET* T992I mutation

## Abstract

Clinical trials for MET inhibitors have demonstrated limited success for their use in colon cancer (CC). However, clinical efficacy may be obscured by a lack of standardisation in *MET* assessment for patient stratification. In this study, we aimed to determine the molecular context in which *MET* is deregulated in CC using a series of genomic and proteomic tests to define *MET* expression and identify patient subgroups that should be considered in future studies with *MET*‐targeted agents. To this aim, orthogonal expression analysis of *MET* was conducted in a population‐representative cohort of stage II/III CC patients (*n* = 240) diagnosed in Northern Ireland from 2004 to 2008. Targeted sequencing was used to determine the relative incidence of *MET* R970C and *MET* T992I mutations within the cohort. *MET* amplification was assessed using dual‐colour dual‐hapten brightfield *in situ* hybridisation (DDISH). Expression of transcribed *MET* and c‐MET protein within the cohort was assessed using digital image analysis on *MET* RNA *in situ* hybridisation (ISH) and c‐MET immunohistochemistry (IHC) stained slides. We found that less than 2% of the stage II/III CC patient population assessed demonstrated a genetic *MET* aberration. Determination of a high *MET* RNA‐ISH/low c‐MET IHC protein subgroup was found to be associated with poor 5‐year cancer‐specific outcomes compared to patients with concordant *MET* RNA‐ISH and c‐MET IHC protein expression (HR 2.12 [95%CI: 1.27–3.68]). The MET RNA‐ISH/c‐MET IHC protein biomarker paradigm identified in this study demonstrates that subtyping of *MET* expression may be required to identify *MET*‐addicted malignancies in CC patients who will truly benefit from MET inhibition.

## Graphical abstract

A population representative study looking at the molecular context in which *MET* is deregulated in colon cancer using a series of genomic and proteomic tests. This study demonstrates the limited number of genetic *MET* aberrations present in a Northern Irish colon cancer cohort and identifies a cancer‐specific poor prognosis *MET* RNA/c‐MET protein subgroup that may be amenable to targeted therapy.

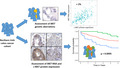

**Abbreviations**
CC: colon cancer
CI: confidence interval
CRC: colorectal cancer
CSS: cancer‐specific survival
DDISH: dual‐colour dual‐hapten brightfield *in situ* hybridisation
Epi700: a population‐representative Northern Irish colon cancer cohort
FFPE: formalin‐fixed paraffin embedded
HR: hazard ratio
IHC: immunohistochemistry
ISH: *in situ* hybridisation
MSI: microsatellite instability

## 1 Introduction

Globally, colorectal cancer (CRC) is the third most common cancer contributing to nearly 10% of all new instances of cancer and cancer‐related deaths [1, 2]. The relative 5‐year survival rate has steadily increased over time due to improvements in the early detection, treatment and management of CRC [1, 3]. In particular, use of targeted therapies have helped to significantly improve survival outcomes in patients with treatment refractory disease. Patient stratification for targeted treatment is often biomarker led in order to determine which patients will receive the most clinical benefit [4]. However, even patients eligible for targeted therapies can go on to develop disease resistance [5]. Therefore, understanding the molecular context in which a biomarker can predict response to treatment is essential for the approval of novel targeted therapies and their companion biomarkers.

*MET*, a proto‐onocogene located on chromosome 7q31.2, has been linked with both lack of phase III clinical trial efficiency and treatment refractory disease following EGFR tyrosine kinase inhibition [5, 6, 7]. *MET* has also been shown to be involved in MEK1/2 inhibitor resistance due to its ability to crosstalk and activate downstream members of the MAPK/ERK pathway [8]. Therefore, *MET* inhibition is a desirable candidate for targeted therapy as MAPK, PI3K‐Akt and STAT pathways are involved in downstream signalling cascades of MET‐addicted malignancies [9]. However, dysregulation of *MET* within the tumour is heterogeneous and its expression can be influenced by genomic aberration, constitutive overexpression, and auto‐ and paracrine stimulation [10, 11]. Consequently, many clinical trials assessing the inhibition of tyrosine kinases fail due to inclusion of patients unlikely to derive benefit from the treatment, resulting from a lack of evidence regarding the genetic and molecular context of *MET* addiction within that cancer type [6].

Evidence of *MET*‐addition is important because novel *MET* inhibitors have only shown efficacy in c‐MET‐addicted cell lines [12]. We have previously demonstrated that poor‐prognosis *MET*‐addicted malignancies have increased *MET* mRNA expression that is not congruent with c‐MET protein expression due to rapid downregulation when c‐MET expression is induced by the presence of HGF [10]. These data show that it may not be possible to reproducibly stratify CRC *MET*‐addicted malignancies when using a single biomarker paradigm. Therefore, there is a clinical need to accurately identify these patients before treatment with MET inhibitors as establishing the best way to stratify patients according to *MET* expression analysis as this may aid patient selection in clinical trials and the development of a companion diagnostic [13]. Herein, the aim of the present study is to establish the relative incidence and prognosis of aberrant c‐MET expression patterns based on mRNA and protein analysis, relative to mutational status and *MET* amplification, using a stage II/III population‐representative colon cancer (CC) cohort. This will be achieved through establishment of a consistent *MET* RNA‐ISH/c‐MET IHC protein scoring method using digital image analysis to enable the identification of a suitably *MET*‐addicted patient subgroup that could potentially benefit from targeted treatment.

## 2 Materials and methods

### 2.1 Patients

All *MET* expression analyses were conducted blinded to patient outcomes on a population‐representative, Northern Irish, CC cohort (Epi700) that was previously identified via the Northern Ireland Cancer Registry and linked with clinicopathological data and mutational status for *BRAF*, *KRAS*, *MET*, *NRAS* and *PIK3CA* [14]. This study was conducted according to the Good Clinical Practice guidelines and the Declaration of Helsinki. All patients provided informed consent for sampling of their tissue as part of their surgical management pathway. Ethical approval for experimental use of these tissue and data was granted through the Northern Ireland Biobank (OREC 21/NI/0019; NIB13/0069, NIB13/0087, NIB13/0088 and NIB15/0168). Briefly, patients diagnosed with a Stage II or Stage III primary adenocarcinoma of the colon, inclusive of ICD codes C18 and C19, between 2004 and 2008 and who underwent surgical resection following diagnosis, were identified and formalin‐fixed paraffin embedded (FFPE) tissue blocks retrieved by the Northern Ireland Biobank for tissue‐based analysis. Chemotherapy regimens given were in line with treatment guidelines in place at the time of diagnosis. Microsatellite instability (MSI) status was previously determined by use of polymerase chain reactions for five mononucleotide repeat markers (BAT‐25, BAT‐26, NR‐21, NR‐24 and MONO‐27) [14]. Mutation status was obtained prior to this study as previously described using a targeted capture panel (ColoCarta Panel v1.0; Agena Bioscience, Hamburg, Germany) to determine mutations in *BRAF* (D594V, V600E, V600K, V600L and V600R), *KRAS* (A59T, G12A, G12C, G12D, G12F, G12R, G12S, G12V, G13D, G61H and Q61L), *MET* (R970C and T992I), *NRAS* (G12C, G12V, G13C, G13V, Q61E and Q61H) and *PIK3CA* (C420R, E542K, E545K, H701P, H1047R, Q546K and R88Q) genes [14, 15]. A patient was considered to be gene mutant if somatic mutation was detected in more than > 10% of reads called for any of the alleles targeted by the panel for that gene. Equivocal/unknown mutation status was reported if the assay reaction failed to report mutation status in any of the alleles assessed for that gene.

### 2.2 Procedures

Standardised operating procedures within the Queen's University Belfast Precision Medicine Centre of Excellence were used for conducting c‐MET immunohistochemistry (IHC), *MET in situ* hybridisation (ISH) assays, digital slide scanning and digital image analysis in the study. All staining and *MET* expression analysis were carried out on tissue sampled from the Epi700 patient cohort in TMA format [16, 17]. TMAs were created as previously described [14]. Briefly, tissue cores with a diameter of 1mm were extracted in triplicate from annotated areas within donor FFPE blocks and inserted into individual recipient blocks using a Beecher manual tissue arrayer (Beecher Instruments Inc, Sun Prairie, WI, USA). Patients were randomised across the TMAs created. The TMAs were then sectioned at 4 μm using a rotary microtome and dried overnight at 37 °C before staining. Senior pathologists (JJ and MST) agreed upon each assay optimisation prior to c‐MET IHC, *MET* DNA and RNA‐ISH staining and digital image analysis. All slides were scanned using the Leica Aperio AT2 (Leica Biosystems, Newcastle, UK) and made available for digital assessment up to 400× magnification.

Immunohistochemistry was carried out on the Ventana^®^ Benchmark XT automated immunostainer (Roche Diagnostics, Basel, Switzerland) using a c‐MET‐specific antibody (Ventana^®^, CONFIRM C‐MET rabbit monoclonal antibody, Clone SP44, Cat. No. 790‐4430). The prediluted antibody was applied neat and incubated for 16 min on the tissue following heat induced epitope retrieval with cell conditioning solution 1 for 60 min. Antibody binding was visualised with DAB chromogen (Ventana^®^, ultraView Universal DAB Detection Kit, Cat. No. 760‐500; Roche Diagnostics). c‐MET IHC protein expression was evaluated by digital image analysis using Definiens TMA module (Definiens Inc., Munich, Germany). IHC stained slides were digitised and an H‐Score obtained for each core by digital image analysis based on the extent and intensity of membranous staining in the malignant epithelium (*H*‐score = 1 × area of low c‐MET expression + 2 × area of moderate c‐MET expression + 3 × area of high c‐MET expression). Up to three TMA cores were available for digital c‐MET IHC protein assessment, therefore, the median *H*‐Score of the cores was taken to generate a c‐MET score per patient.

The Leica Bond RX automated immunostainer (Leica Biosystems) was used to carry out RNA‐ISH using the RNAscope assay to produce robust staining [18]. RNA‐ISH staining was conducted using a probe targeting *MET* [ACD RNAScope^®^, LS 2.5 Probe‐ Hs‐MET‐FL (NM_000245.2, 175‐6505), Cat. No. 423108] and visualised with chromogenic detection using DAB (ACD RNAScope^®^, 2.5 HD Reagent kit—brown from, ACD, Cat. No. 322300). RNA‐ISH stained slides were digitised and regions of interest in the tumour epithelium digitally evaluated using the assisted scoring software halo (Indica Labs, Albuquerque, NM, USA). Digital image analysis enabled precise quantitation of RNA‐ISH tissue data which included the total tumour area and the total probe area within the core. Nuclear segmentation and cell detection were not conducted in this study as sample pre‐treatment required by the automated RNAscope assay led to loss of distinct cellular morphology in the tissue. Hence, we calculated an additional value for analysis representing the number of probe signals per micrometre squared of tumour tissue. This score was created by dividing the values for the total positive probe area by the total tumour area. Similar to c‐MET IHC protein expression analysis, the median probe area across the three TMA cores was taken to generate a *MET* RNA‐ISH score per patient.

DNA‐ISH was carried out on the Ventana^®^ Benchmark XT automated immunostainer (Roche Diagnostics). DNA‐ISH staining was conducted using the dual‐colour dual‐hapten brightfield ISH method (DDISH) for evaluation of *MET* amplification using the *MET* DNP Probe (Ventana^®^, *MET* DNP Probe, Cat. No. 760‐1228) and Chromosome 7 DIG Probe (Ventana^®^, Chromosome 7 DIG Probe, Cat. No. 760‐1219) as per manufacturer's instructions. Chromogenic detection of the *MET* probe was visualised with silver ISH DNP detection (Ventana^®^, ultraView SISH DNP Detection Kit, Cat. No. 760‐098) while CHR7 probe was visualised with red ISH DIG detection (Ventana^®^, ultraView Red ISH DIG Detection Kit, Cat. No. 760‐505). The ratio between the black *MET* and red CHR7 probe signals was determined by manual light microscopy at 400× magnification. Twenty tumour cells were evaluated, and an average score was determined in potentially amplified cores. The amplification cut‐off was set at a MET/CHR7 ratio of three. Each patient was designated as *MET* amplified or nonamplified via DDISH, if one of the three TMA cores was determined as amplified during DDISH evaluation.

### 2.3 Statistical analysis

All statistical analysis was performed in r version 3.6.1 (R Foundation, Vienna, Austria). Kaplan–Meier plots and log‐rank *P* values were used to illustrate the 5‐year cancer‐specific survival (CSS) between dichotomised low and high *MET* patient groups. CSS was the time between diagnosis and death specifically caused by CRC as determined by ICD cause of death codes C18, C19, C20 and/or C26. Data were right‐censored for patients with incomplete survival information and in patients with greater than 5‐year survival. Univariate and multivariate analyses were conducted using the Cox proportional hazard method to generate hazard ratios (HR) with 95% confidence intervals (CI). Multivariable models were adjusted for age, sex, UICC TNM stage, MSI status and whether adjuvant chemotherapy was received. Sensitivity analysis of multivariable models was conducted on this dataset by left‐censoring patients with a follow‐up of 6 months or less. Association of continuous and categorical variables between groups was assessed using either ANOVA or Pearson's chi‐square test for independence when appropriate. Scatter plots were used to visualise the relationship between orthogonal *MET* biomarker assays and correlations reported using Spearman's Rho.

This study was conducted in accordance to REporting recommendations for tumour MARKer prognostic studies (REMARK) [19, 20]. The purpose of this biomarker study was to evaluate the prognostic significance of orthogonal expression of *MET* in relation to survival within a retrospective, population‐representative cohort of CC. The reporting standards of the current study fulfil these recommendations.

## 3 Results

### 3.1 Patients

**Fig. 1:**
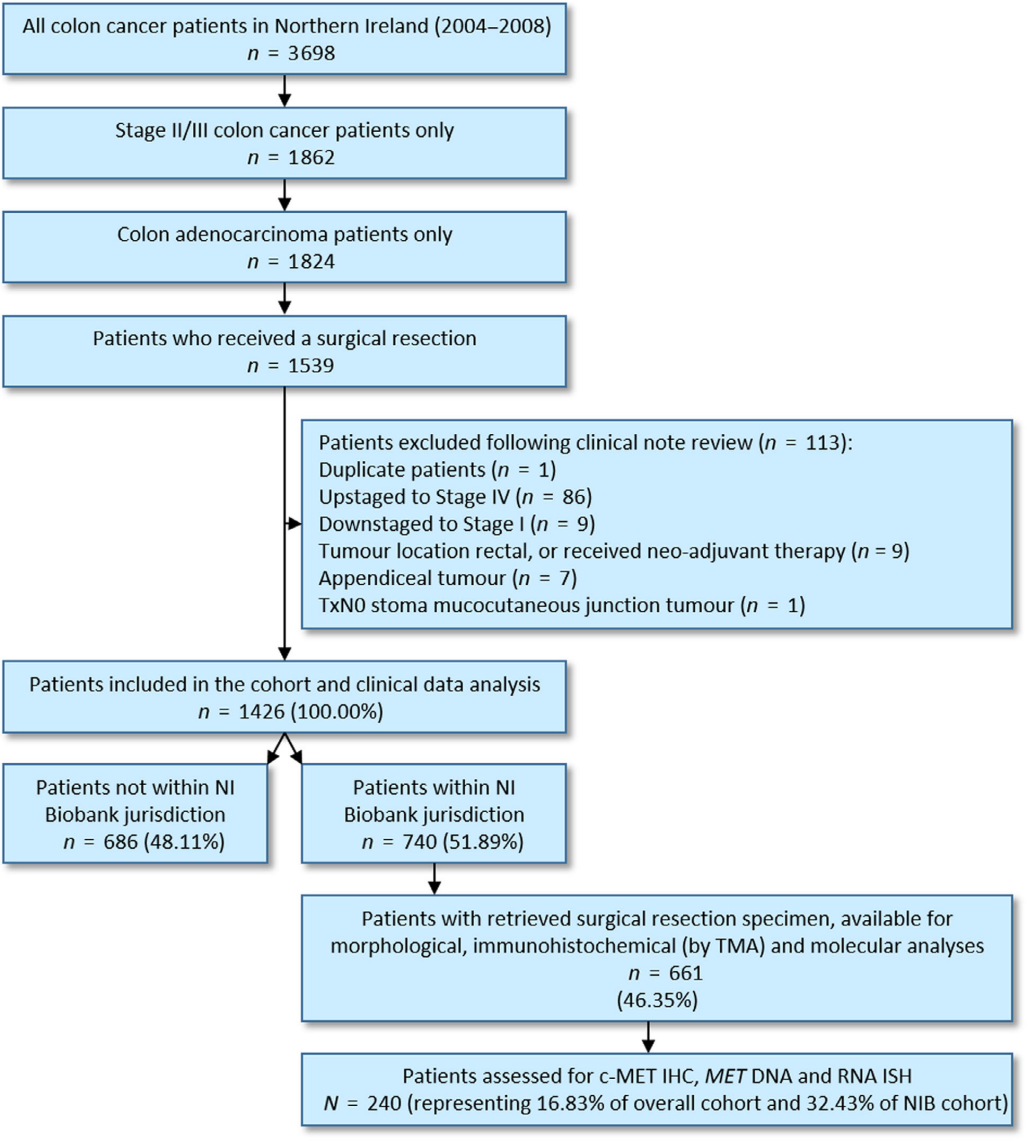
STROBE diagram for the selection of a population‐representative stage II/III colon cancer cohort. Adapted from Gray *et al*. [14].

Orthogonal *MET* expression analysis was carried out in 240 of the original cohort (32.43%) (Fig. 1). Full clinicopathological and biomarker data were available in these patients. Pearson's chi‐squared tests revealed no significant difference between the original and reduced cohorts for all clinical factors (*P* > 0.0500; Table 1). The cohort of patients assessed for *MET* expression analysis (*n* = 240) was therefore assumed to be representative of the retrieved population‐representative, Northern Irish, Stage II/III CC cohort (*n* = 661) in terms of age, sex, stage, MSI status and chemotherapy administration in the current study.

**Table 1:** Comparison of baseline characteristics. Data are presented as number of patients (%). Differences in patient characteristics between the study cohorts using ANOVA and Pearson's chi‐squared test for continuous and categorical variables, respectively.

	Study cohort (*n* = 240)	Epi700 (*n* = 661)	*P* value
Median age (interquartile range)	72 (63–78)	72 (64–79)	0.9470
Age			
< 70	102 (42.50%)	282 (42.66%)	0.9652
70+	138 (57.70%)	379 (57.34%)	
Sex			
Male	133 (55.42%)	358 (54.16%)	0.7378
Female	107 (44.58%)	303 (45.84%)	
UICC TNM stage			
II	135 (56.25%)	394 (59.61%)	0.3657
III	105 (43.75%)	267 (40.39%)	
MSI status			
Stable	166 (69.17%)	471 (71.26%)	0.6717
High	50 (20.83%)	136 (20.57%)	
Missing	24 (10.00%)	54 (8.17%)	
Adjuvant chemotherapy			
No	167 (69.58%)	475 (71.86%)	0.5043
Yes	73 (30.42%)	186 (28.14%)	

### 3.2 MET expression analysis

**Fig. 2:**
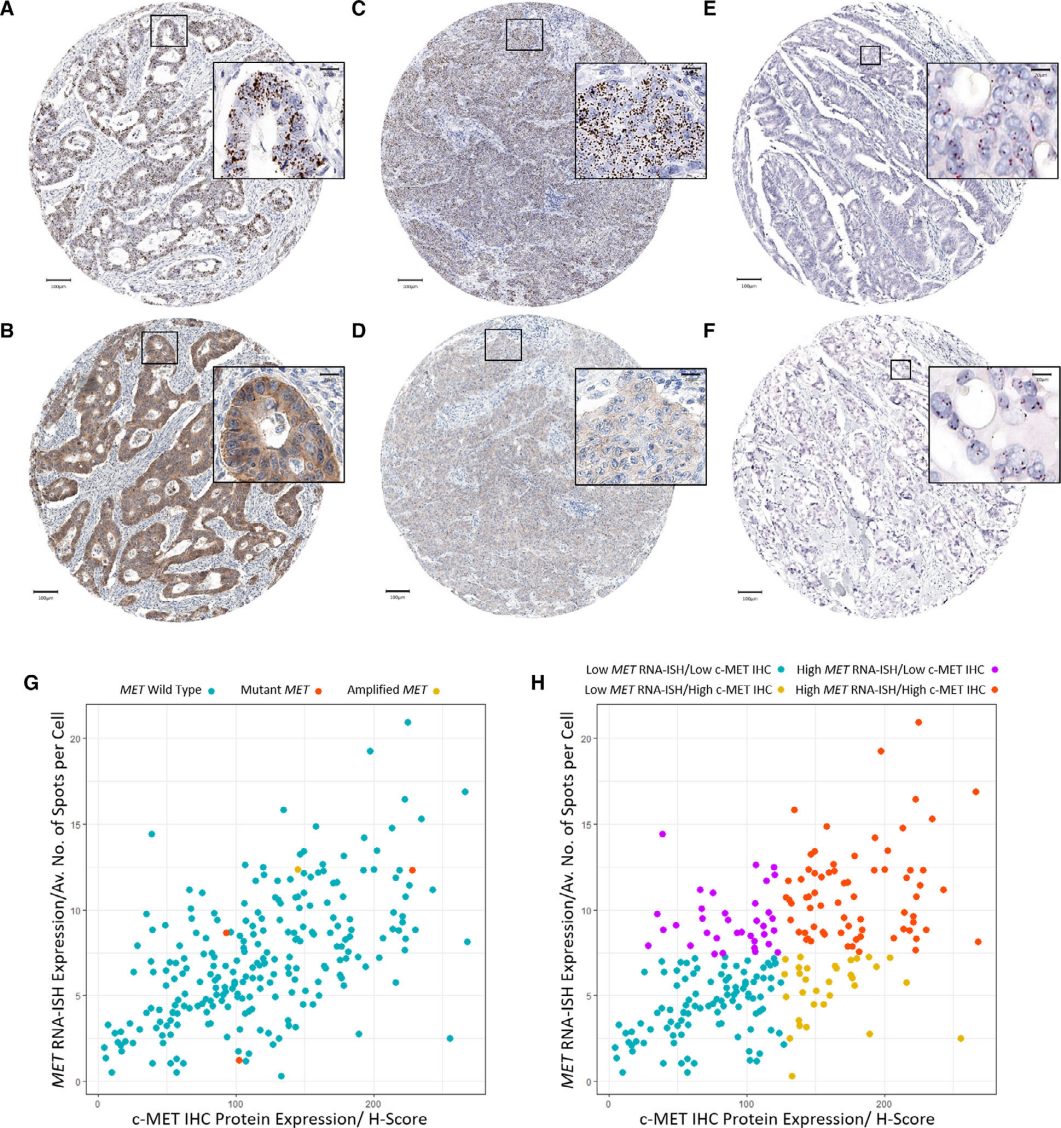
Representative images displayed at 10× and 40× magnification (scale bars = 100 and 20 µm, respectively) from patients with concordant *MET* RNA‐ISH (A)/c‐MET IHC protein expression (B) (*n* = 1), nonconcordant high *MET* RNA‐ISH (C)/low c‐MET IHC protein expression (D) (*n* = 1), nonamplified DDISH indicating no *MET* amplification present as seen in the majority (*n* = 239) of the patient cohort (E) and the patient identified with amplified DDISH indicating *MET* amplification was present (F). Scatter plot demonstrating the relationship *MET* RNA‐ISH/c‐MET IHC protein expression in relation to *MET* mutation or amplification status in the study cohort (*n* = 240) (G). Scatter plot demonstrating the split of data (*n* = 240) into *MET* RNA‐ISH/c‐MET IHC protein expression subgroups (H).

*MET* amplification, *MET* mutation, *MET* RNA‐ISH and c‐MET IHC protein expression levels were assessed across the patient cohort as described. Variable *MET* RNA‐ISH and c‐MET IHC protein expression levels were observed within the cohort (Fig. 2A–D). In contrast, DDISH analysis determined that *MET* amplification was present in only one patient (0.42%) of the study cohort with a c‐MET and CHR7 ratio of 4.03 (Fig. 2E,F). Further, only three patients (1.25%) were identified as having either R970C or T992I mutations in the *MET* gene. *MET* RNA‐ISH expression demonstrated a moderate positive correlation with increasing c‐MET IHC protein expression (*R* ^2^ = 0.56; *P* < 0.0001). The patient with *MET* amplification were found to cluster near patients with increased levels of both *MET* RNA‐ISH and c‐MET IHC protein expression, while no clustering was observed in patients with *MET* mutations (Fig. 2G). Dichotomisation of *MET* expression was defined by ROC curve analysis for *MET* RNA‐ISH and c‐MET IHC protein expression against 5‐year survival CSS [21]. The optimal cut‐off for *MET* RNA‐ISH expression was determined to be an average number of Spots per Cell of 7.350, while for c‐MET IHC protein expression, it was an *H*‐Score of 127.105. To account for possible post‐transcriptional events of potential clinical relevance, dichotomised *MET* RNA‐ISH and c‐MET IHC protein results were combined in order to determine the proportion of patients in the population who had concordant and discordant *MET* RNA‐ISH and c‐MET IHC protein expression. Concordant *MET* RNA‐ISH and c‐MET IHC protein expression was present in 173 (72.08%) patients while nonconcordance was observed in 32 (13.33%) and 35 (14.58%) of the study cohort in patients with low *MET* RNA‐ISH, high c‐MET IHC protein expressing tumours and in high *MET* RNA‐ISH and low c‐MET IHC protein expressing tumour, respectively (Fig. 2H). Pearson's chi‐squared tests revealed no significant difference between the concordant and discordant *MET* RNA‐ISH and c‐MET IHC protein expressing tumours for clinical–pathological variables and mutations present (*P* > 0.0500; Table 2).

**Table 2:** Comparison of baseline characteristics and mutation status, according to MET RNA‐ISH and c‐MET IHC protein expression subgroups. Data are presented as number of patients (%). Differences compared to RNA/IHC subgroups using Pearson's chi‐squared test for categorical variables.

	Concordant *MET* RNA‐ISH/c‐MET IHC (*n* = 173)	Low *MET* RNA‐ISH/High c‐MET IHC (*n* = 32)	High *MET* RNA‐ISH/Low c‐MET IHC (*n* = 35)	*P* value
Age at diagnosis				
< 70	75 (43.35%)	14 (43.75%)	13 (37.14%)	0.7855
70+	98 (56.65%)	18 (56.25%)	22 (62.86%)	
Sex				
Male	93 (53.76%)	18 (56.25%)	22 (62.86%)	0.6108
Female	80 (46.24%)	14 (43.75%)	13 (37.14%)	
UICC TNM stage				
II	97 (56.07%)	22 (68.75%)	16 (45.71%)	0.1643
III	76 (43.93%)	10 (31.25%)	19 (54.29%)	
MSI status				
Stable	117 (67.63%)	23 (71.88%)	26 (74.29%)	0.8860
High	37 (21.39%)	6 (18.75%)	7 (20.00%)	
Missing	19 (10.98%)	3 (9.38%)	2 (5.71%)	
Adjuvant chemotherapy				
No	120 (69.36%)	22 (68.75%)	25 (71.43%)	0.9653
Yes	53 (30.64%)	10 (31.25%)	10 (28.57%)	
*BRAF* status				
Wild‐type	148 (85.55%)	28 (87.50%)	27 (77.14%)	0.5595
Mutant	23 (13.29%)	4 (12.50%)	8 (22.86%)	
Equivocal/Unknown	2 (1.16%)	0 (0.00%)	0 (0.00%)	
*KRAS* status				
Wild‐type	114 (65.90%)	16 (50.00%)	21 (60.00%)	0.2150
Mutant	59 (34.10%)	16 (50.00%)	14 (40.00%)	
Equivocal/Unknown	0 (0.00%)	0 (0.00%)	0 (0.00%)	
*MET* status				
Wild‐type	171 (98.84%)	32 (100.00%)	34 (97.14%)	0.5628
Mutant	2 (1.16%)	0 (0.00%)	1 (2.86%)	
Equivocal/Unknown	0 (0.00%)	0 (0.00%)	0 (0.00%)	
*NRAS* status				
Wild‐type	163 (94.22%)	31 (96.88%)	35 (100.00%)	0.3006
Mutant	10 (5.78%)	1 (3.13%)	0 (0.00%)	
Equivocal/Unknown	0 (0.00%)	0 (0.00%)	0 (0.00%)	
*PIK3CA* status				
Wild‐type	143 (82.66%)	24 (75.00%)	26 (74.29%)	0.7318
Mutant	26 (15.03%)	7 (21.88%)	8 (22.86%)	
Equivocal/Unknown	4 (2.31%)	1 (3.13%)	1 (2.86%)	

### 3.3 MET survival analysis

**Fig. 3:**
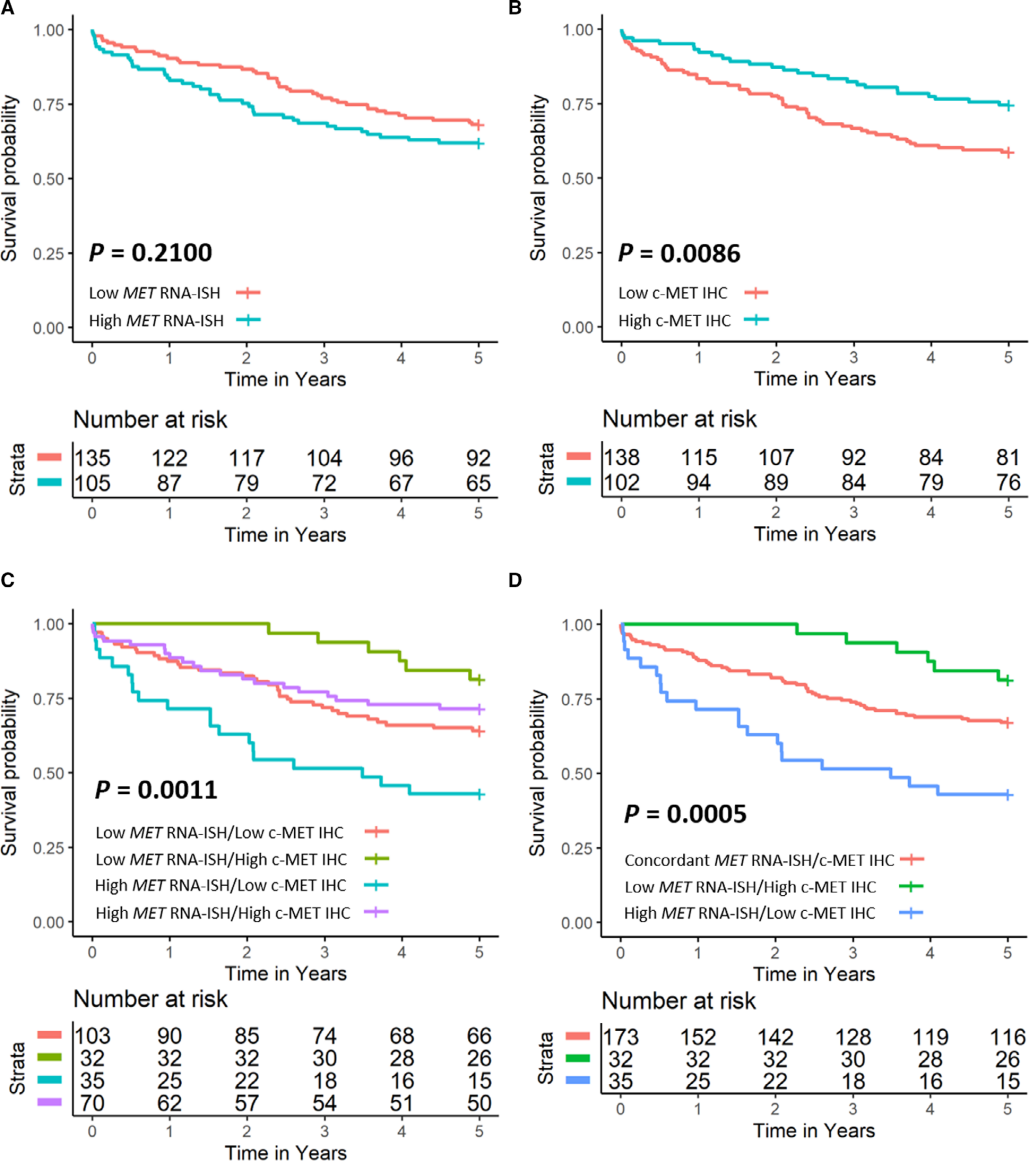
Kaplan–Meier estimates of 5‐year CSS for dichotomised *MET* RNA‐ISH expression (A), c‐MET IHC protein expression (B) and combined *MET* RNA‐ISH/c‐MET IHC protein expression (C, D). Global differences in survival curves were compared through use of the log‐rank test.

Kaplan–Meier plots of dichotomised *MET* RNA‐ISH and c‐MET IHC protein expression against patient outcomes demonstrated that c‐MET IHC protein expression was a more useful assay for determining patient CSS than assessment of *MET* RNA‐ISH (*P* = 0.0086 for c‐MET IHC protein vs. *P* = 0.2100 for *MET* RNA‐ISH; Fig. 3A,B). Interestingly, joint assessment of *MET* RNA‐ISH and c‐MET IHC protein expression by combination of dichotomised assay results found that nonconcordant *MET* RNA‐ISH and c‐MET IHC expression levels were associated with distinct survival outcomes when compared to concordant *MET* RNA‐ISH and c‐MET IHC protein expression levels (*P* = 0.0011; Fig. 3C). No difference in CSS was observed when high vs. low concordant patients were considered, and therefore, concordant cases were collapsed into a single class for CoxPH regression analysis (*P* = 0.0005; Fig. 3D).

Multivariable analysis found nonconcordant *MET* RNA‐ISH and c‐MET IHC protein expression to be an independent predictor of CSS when adjusted for age, sex, UICC TNM stage, MSI status and use of adjuvant chemotherapy (Table 3). Patients with nonconcordant high *MET* RNA‐ISH and low c‐MET IHC protein expression were twice as likely to experience a colorectal‐specific death within 5 years of diagnosis compared to patients with concordant *MET* RNA‐ISH and c‐MET IHC protein expression (HR 2.12 [95% CI: 1.27–3.55]; *P* = 0.0042). Whereas no significant difference in survival was seen in patients with nonconcordant low *MET* RNA‐ISH, high c‐MET IHC protein expression compared to patients with concordant *MET* RNA‐ISH and c‐MET IHC protein expression (HR 0.52 [95% CI: 0.22–1.22]; *P* = 0.1316). Sensitivity analysis confirmed that the relative risk of mortality was not influenced by cancer‐specific deaths of 6 months or less in the study cohort (Table 4). Survival analysis was not conducted for *MET* amplification or mutation due to small numbers (*n* = 1 and *n* = 3, respectively).

**Table 3:** Univariate and multivariable analysis for 5‐year CSS in study patients. Data are HRs (95% CI) and corresponding *P* values. Models were mutually adjusted for each variable included in the table using pairwise comparison for the reference category in each covariate.

	Univariate	*P* value	Multivariable	*P* value
Age				
< 70 : 70+	1.53 (0.97–2.40)	0.0663	1.08 (0.66–1.77)	0.7458
Sex				
Male : Female	0.74 (0.48–1.15)	0.1810	0.74 (0.48–1.16)	0.1932
UICC TNM stage				
II : III	2.10 (1.36–3.26)	0.0008	2.97 (1.85–4.75)	< 0.0001
MSI status				
Stable : High	0.58 (0.30–1.1)	0.0935	0.59 (0.31–1.13)	0.1092
Stable : Missing	1.49 (0.88–2.77)	0.2057	1.62 (0.87–3.03)	0.1292
Adjuvant chemotherapy				
No : Yes	0.51 (0.30–0.88)	0.0144	0.33 (0.18–0.59)	0.0003
*MET* RNA‐ISH/c‐MET protein subgroup				
Concordant *MET* RNA‐ISH/c‐MET IHC : Low *MET* RNA‐ISH/High c‐MET IHC	0.48 (0.21–1.12)	0.0900	0.52 (0.22–1.22)	0.1316
Concordant *MET* RNA‐ISH/c‐MET IHC : High *MET* RNA‐ISH/Low c‐MET IHC	2.12 (1.32–3.68)	0.0024	2.12 (1.27–3.55)	0.0042

**Table 4:** Univariate and multivariable sensitivity analysis for 5‐year CSS in study patients. Data are HRs (95% CI) and corresponding *P* values. Models were mutually adjusted for each variable included in the table using pairwise comparison for the reference category in each covariate.

	Univariate	*P* value	Multivariable	*P* value
Age				
< 70 : 70+	1.14 (0.70–1.88)	0.6010	0.93 (0.54–1.59)	0.7799
Sex				
Male : Female	0.69 (0.42–1.15)	0.1580	0.70 (0.42–1.16)	0.1631
UICC TNM stage				
II : III	1.80 (1.10–2.95)	0.0168	2.42 (1.40–4.16)	0.0015
MSI status				
Stable : High	0.46 (0.21–1.01)	0.0521	0.49 (0.22–1.09)	0.0786
Stable : Missing	1.41 (0.69–2.88)	0.3399	1.51 (0.74–3.09)	0.2565
Adjuvant chemotherapy				
No : Yes	0.71 (0.41–1.23)	0.2180	0.45 (0.23–0.85)	0.0147
*MET* RNA‐ISH/c‐MET protein subgroup				
Concordant *MET* RNA‐ISH/c‐MET IHC : Low *MET* RNA‐ISH/High c‐MET IHC	0.61 (0.26–1.42)	0.2494	0.63 (0.27–1.49)	0.2919
Concordant *MET* RNA‐ISH/c‐MET IHC : High *MET* RNA‐ISH/Low c‐MET IHC	2.13 (1.17–3.90)	0.0136	2.09 (1.14–3.83)	0.0172

## 4 Discussion

The *MET* pathway is frequently dysregulated in cancer and acquired genomic aberrations can lead to treatment refractory disease with EGFR tyrosine kinase inhibitors [5, 7]. MET inhibitors have been developed to impede aberrant enzymatic activity of c‐MET [22]. Therapeutic use of small molecule MET inhibitors has been approved for use in medullary thyroid, renal cell and subsets of nonsmall cell lung carcinomas, but have failed to demonstrate clinical efficacy for other cancer types including CRC due to inappropriate patient selection [6]. Both genomic and tissue‐based assays have been used clinically to predict a patient's likelihood of response to MET inhibition in CRC, but a lack of population‐based evidence to interpret the prognostic outcomes of aberrant c‐MET activity in CRC means there is little evidence to support their use [6, 13]. It has been previously shown that HGF induced c‐MET protein can be constitutively overexpressed and undergo rapid downregulation while the MET transcript remained unaltered [10]. We therefore believe that assessment of both mRNA and protein expression is important because the rapid turnover of c‐MET cannot be accurately reflected by assessment of the protein alone in fixed tissue analyses. The analysis presented in the study indicates that assessment of MET mRNA from the tumour bulk is useful but that it is only whenever assessment of the protein is also considered do you see patients with genuinely MET‐addicted malignancy. This is the first study to contextualise *MET* expression in CC through use of orthogonal technologies for *MET* quantification in a population‐representative cohort. This study demonstrates that c‐MET overexpression arises most often due to a relative increase in RNA expression in stage II/III CC. Importantly, this study identifies subgroups of patients with discordant *MET* RNA‐ISH/c‐MET IHC protein expression who may benefit from dual testing with RNA‐ISH and IHC and that should be considered in future clinical trials using MET inhibitors.

This study found that the incidence of *MET* genomic aberration affected less than 2% of stage II and stage III CCs diagnosed in Northern Ireland, with *MET* amplification and mutation independently occurring in 0.42% and 1.25% of patients. *MET* RNA‐ISH and c‐MET IHC protein expression was found to demonstrate a moderate‐positive relationship in the absence of genetic aberration influencing relative c‐MET expressed from the tumour. In patients with genetic aberrations present, *MET* amplification was found to be associated with increased *MET* RNA‐ISH and c‐MET IHC protein expression, while *MET* mutations appear to randomly influence overall c‐MET expressed. However, the number of patients with *MET* aberrations present in this cohort are too small to draw firm conclusions from the data. In a third of patients assessed for *MET* expression analysis, there was a lack of agreement in dichotomised low and high groups representing *MET* RNA‐ISH and c‐MET IHC protein expression, with nonconcordance demonstrated in 27.91% of tumours assessed. Of these, only patients with high *MET* RNA‐ISH/low c‐MET IHC protein expressing tumours were found to be twice as likely to die of a colorectal‐specific death in 5 years. These patients represented 14.58% of the overall study population. No significant difference in survival was observed in patients with low *MET* RNA‐ISH/high c‐MET IHC protein expressing tumours compared to tumours with concordant *MET* RNA‐ISH/c‐MET IHC protein expression. However, these patients represent 13.33% of the study population who exhibit evidence of enhanced dimerization with tyrosine kinase receptors and upregulated production of the c‐MET IHC protein. Importantly, concordant high *MET* RNA‐ISH/c‐MET IHC protein expressing tumours demonstrate no significant survival compared to the low *MET* RNA‐ISH/c‐MET IHC protein expressing patients. This demonstrates that while *MET* is dysregulated in these patients, it is unlikely to be the oncogenic pathway driving tumorigenesis in those patients. Rather, it is the patient subgroup with high *MET* RNA‐ISH/low c‐MET IHC protein expressing tumours that would most likely benefit from MET inhibition.

This study found lower than expected incidence of *MET* genomic aberrations. MET amplification was only found to have an incidence of 0.4% when assessed in stage II/III CC, which was significantly lower than the 1.9–2.2% reported elsewhere [23, 24, 25]. In contrast to this study, which was conducted in stage II/III CC, relative incidence of *MET* amplification in large studies were reported on metastatic CRC only [23]. Supporting this, Jardim *et al*. [24] reported that incidence of *MET* amplification was more likely to occur in patients with metastatic disease and may contribute to the lower than expected incidence of *MET* amplification present in the current study. Further, incidence of *MET* mutation reported in the current study was also significantly below the expected 2–5% demonstrated in the literature [23]. This was not an unexpected finding as the choice to use a target capture panel to assess *MET* mutation instead of whole genome sequencing restricted the number of *MET* mutations that could be called to two single point mutations. Our findings of 1% incidence in *MET* R970C and T992I point mutations are in line with Tyner *et al*. [26] who looked at incidence of these specific mutations in CRC. Lack of whole genome sequencing meant we also did not assess *MET* exon 14 skipping in the current study, and however, this has been previously shown to not occur in CRC [23].

## 5 Conclusions

In conclusion, MET inhibitors are used to target c‐MET expressing tumours. Through use of population research, this study demonstrates that in the absence of genomic aberration via either *MET* gene amplification or *MET* R970C and T992I point mutations, nonconcordant patterns of *MET* expression are associated with 5‐year CSS in CC. The impact of *MET* expression subgroups on efficacy of MET inhibition was not considered in the current study design and warrants investigation in future studies.

## Conflict of interest

Dr MS‐T has recently received honoraria for advisory work in relation to the following companies: Incyte, MindPeak, QuanPathDerivatives and MSD. He is part of academia‐industry consortia supported by the UK government (Innovate UK). Dr JAJ is also part of the academia‐industry consortia supported by the UK government (Innovate UK). These declarations of interest have no relationship with the submitted publication. All other authors declare no competing interests.

## Author contributions

All authors contributed to interpretation of data and writing of the manuscript. RHW, SVS, JAJ and MS‐T were involved in study conception and design. SGC, SM, MPH, VB, AVP and SMcQ contributed to data acquisition. SGC, SM and VB were involved in data analysis.

### Peer Review

The peer review history for this article is available at https://publons.com/publon/10.1002/1878‐0261.13089.

## Data Availability

Data are held within the Northern Ireland Biobank and are available on application.
